# Sustaining Maternal and Neonatal Tetanus Elimination (MNTE) in countries that have been validated for elimination – progress and challenges

**DOI:** 10.1186/s12889-022-13110-2

**Published:** 2022-04-08

**Authors:** Nasir Yusuf, Robert Steinglass, Francois Gasse, Azhar Raza, Bilal Ahmed, Diana Chang Blanc, Ahmadu Yakubu, Christopher Gregory, Rania A. Tohme

**Affiliations:** 1 Department of Immunization, Vaccines and Biologicals, World Health Organization, Avenue Appia 20, 1211 Geneva, Switzerland; 2 Independent Entity, Marshall, NC USA; 3 Independent Entity, San Antoni, Spain; 4 Program Division, United Nations Children Fund (UNICEF), New York, USA; 5 Global Immunization Division, Centers for Disease Control and Prevention, Atlanta, GA USA

**Keywords:** Tetanus, Vaccination, Disease elimination, Vaccine preventable diseases, Sustainability, Booster doses

## Abstract

**Background:**

As of October 2021, 47 (80%) of the 59 countries, identified at highest risk for Maternal and Neonatal Tetanus (MNT), had been validated for elimination. We assessed sustainability of MNT elimination (MNTE) in 28 countries that were validated during 2011‒2020.

**Methods:**

We assessed the attainment of the following MNTE sustainability indicators: 1) ≥ 90% coverage with three doses of Diphtheria-Tetanus-Pertussis vaccine (DTP3) among infants < 1 year, 2) ≥ 80% coverage with at least two doses of tetanus toxoid-containing vaccine (TTCV2 +) among pregnant women, 3) ≥ 80% protection at birth (PAB), 4) ≥ 70% skilled birth attendance (SBA), and 4) ≥ 80% first (ANC1) and fourth antenatal care (ANC4) visits. We assessed the introduction of TTCV booster doses. Data sources included the 2020 WHO /UNICEF Joint Reporting Forms, and the latest Demographic and Health Survey (DHS) or Multi-Indicator Cluster Surveys (MICS) for each country, if available. We reviewed literature and used DHS/MICS data to identify barriers to sustaining MNTE.

**Results:**

Of 28 assessed countries, 7 (25%) reported ≥ 90% DTP3 coverage, 4 of 26 (16%) reported ≥ 80% TTCV2 + coverage, and 23 of 27 (85%) reported ≥ 80% PAB coverage. Based on DHS/MICS in 15 of the 28 countries, 10 (67%) achieved ≥ 70% SBA delivery, 13 (87%) achieved ≥ 80% ANC1 visit coverage, and 3 (20%) ≥ 80% ANC4 visit coverage. We observed sub-optimal coverage in many countries at the subnational level. The first, second and third booster doses of TTCV respectively have been introduced in 6 (21%), 5 (18%), and 1 (4%) of 28 countries. Only three countries conducted post-MNTE validation assessments. Barriers to MNTE sustainability included: competing program priorities, limited resources to introduce TTCV booster doses and implement corrective immunization in high-risk districts and socio-economic factors.

**Conclusions:**

Despite good performance of MNTE indicators in several countries, MNTE sustainability appears threatened in some countries. Integration and coordination of MNTE activities with other immunization activities in the context of the Immunization Agenda 2030 lifecourse vaccination strategy such as providing tetanus booster doses in school-based vaccination platforms, during measles second dose and HPV vaccination, and integrating MNTE post-validation assessments with immunization program reviews will ensure MNTE is sustained.

## Background

Clostridium tetani (C. tetani), the causative agent of tetanus is widely found in soil and can easily enter the body through contaminated wounds, exposing unimmunized individuals to tetanus. Unclean delivery and umbilical cord care practices are the main causes of maternal and neonatal tetanus [1]. In 1989 the World Health Assembly (WHA) [2] endorsed the resolution on Neonatal Tetanus Elimination and in 1999, the global initiative was relaunched as maternal and neonatal tetanus elimination (MNTE) targeting 59 priority countries that were considered at high risk for maternal and neonatal tetanus (MNT) [3]. MNTE is defined as having less than one neonatal tetanus (NT) case per 1000 livebirths (< 1 NT / 1000 live births) in every district annually [4]. The strategies for elimination include: 1) vaccinating ≥ 80% of pregnant women in all districts with 2 doses of tetanus toxoid containing vaccine (TTCV2 +), 2) targeting ≥ 80% of women of reproductive age (WRA) aged 15‒49 years in high-risk districts with TTCV through rounds of supplementary immunization activities (SIAs), 3) promoting clean births and umbilical cord care so that ≥ 70% of women having access to skilled birth attendance (SBA), and 4) strengthening NT surveillance including case investigation and response [5]. As of October 2021, 47 (80%) of the 59 priority countries have been validated to have achieved MNTE [6].

Once countries have been validated for MNTE, WHO recommends four strategies to sustain elimination [7, 8]. These include: 1) providing three primary doses of TTCV during infancy and three booster doses at 12 – 23 months, 4 – 7 years and 9 – 15 years respectively, 2) checking tetanus vaccination status during antenatal care and providing TTCV if needed to pregnant women to ensure tetanus protection at birth (PAB), 3) promoting clean delivery and umbilical cord care practice through increased access to skilled birth attendants (doctors, nurses and midwives), and 4) maintaining strong neonatal tetanus (NT) surveillance. Following validation of elimination, WHO recommends that validated countries should conduct post-validation assessment every five years to assess their MNTE sustainability status [8]. It also recommends countries conduct annual NT risk analyses to identify and to take corrective actions in high-risk districts [8, 9].

During 2011–2020, 28 countries were validated for MNTE. Most of these countries had routine immunization systems with suboptimal performance and relied on the recommended WHO “high-risk” approach [10] to achieve MNTE by using TTCV SIAs to vaccinate WRA in high-risk districts. Countries that could not have achieved MNTE without implementing the high-risk approach are particularly at risk of regressing to a situation where MNT could reemerge as a public health concern if strategies for maintaining MNTE are not sustained.

We assessed the progress and challenges to MNTE sustainability in the 28 countries that were validated during 2011–2020 and identified options for enhancing the implementation of the recommended MNTE sustainability strategies.

## Methods and materials

We compiled MNTE sustainability performance indicators from various data sources. We assessed protection against tetanus through immunization of women and infants in all 28 countries by compiling coverage with three doses of diphtheria-tetanus-pertussis vaccine (DTP3) among children aged < 12 months; coverage with TTCV2 + among pregnant women using official country estimates reported to WHO / UNICEF Joint Reporting Form (JRF) in 2020 [11]; and percentage of infants protected at birth (PAB) against tetanus, assessed based on the timing since the last dose and number of doses of TTCV received by the mother obtained from the 2020 WHO estimates [12].

To estimate access to deliveries performed under hygienic conditions and antenatal care in 15 (54%) of the 28 countries that conducted demographic and health surveys (DHS) or multiple indicator cluster survey (MICS) post-MNTE validation, we used data from the most recent DHS/MICS [13, 14] to compile data about women aged 15–49 years who reported a live birth during the last five years preceding the survey to estimate 1) percentage of births with SBA, 2) percentage births with health facility deliveries (HFD), and 3) percentage of women who attended one antenatal care (ANC1) visit, and four antenatal care (ANC4) visits.

We assessed tetanus protection across the life-course in the 28 countries by compiling information on the status of introduction of TTCV booster doses, second dose of measles containing vaccine (MCV2), and human papillomavirus vaccine (HPV), and on the existence of school-based vaccination programs (SVP), from the 2020 WHO / UNICEF Joint Reporting Forms (JRF) [11]. We reviewed the countries’ income status [15] and eligibility for Gavi support for other vaccines and initiatives [16]. We compared the availability of SVP and Gavi eligibility with the status of introduction of one, two and three TTCV booster doses and MCV2 and HPV vaccine.

We also compiled the demographic (total population, number of surviving infants, WRA, and pregnant women) and economic characteristics (income status) of the 28 countries using WHO and World Bank data sources [11, 15].

In view of the sup-optimal NT surveillance in most of the countries and well-known under-reporting of NT cases, we were unable to assess the performance of countries for NT incidence.

A scoping review of the literature, DHS/MICS data review and reports of post-validation assessments from the five countries (Algeria, Cameroon, Djibouti, Indonesia and, Timor-Leste) that conducted the WHO recommended post-MNTE validation assessments provided insights into the barriers to sustaining MNTE.

### Data analysis

Using the data sources mentioned above, we estimated the percentage of countries that achieved 1) ≥ 90% DTP3 coverage among children less than 12 months of age, 2) ≥ 80% TTCV2 + coverage among pregnant women, 3) ≥ 80% of infants protected at birth (PAB) against tetanus, 4) ≥ 70% coverage with SBA, 5) ≥ 70% coverage with HFD, and 6) ≥ 80% ANC1 and ANC4 visits among women aged 15–49 years.

We compared the percentage coverage between TTCV2 + and PAB to describe the discrepancies in coverage that usually exist between these two methods of computing tetanus protection coverage. PAB is considered a more accurate measure of overall protection against tetanus since it considers the total number of doses of TTCV that a woman has received in her lifetime while TTCV2 + only takes into consideration doses taken during the current pregnancy [8].

We used DHS/MICS results in the 15 countries with recent survey data to compile the minimum and maximum coverage of MNTE indicators (TTCV2 + , PAB, SBA, and HFD) by the second subnational level (region/province or State) to assess equity in maintenance of MNTE within countries.

### Ethical consideration

WHO/UNICEF JRF data are aggregate data reported by countries on a yearly basis to WHO and are publicly available [11]. DHS and MICS are standard household surveys conducted in accordance with guidelines of implementing countries, DHS surveys are reviewed and approved by the ICF Institutional Review Board and usually by in-country review boards; publicly available DHS and MICS datasets are anonymized [13, 14].

## Results

### Country characteristics

The 28 countries validated for MNTE during 2011–2020 had a combined 2020 estimated population of approximately 3.7 billion (nearly half the world population), with China and India accounting for over 70% of the population of these countries. There were an estimated 73 million surviving infants that were targeted for DTP3 and 62 million pregnant women (excluding China and Iraq, where there were no data) targeted for TTCV (Table 1). Of the 28 countries, 22 (79%) countries were classified as low-income countries (LICs) or lower-middle income countries (LMICs) and were eligible for support from Gavi, the Vaccine Alliance. All LICs, except Haiti, were African countries, and 17 (90%) of the 19 African countries were eligible for Gavi financial support (Table 1).

**Table 1:** Demographic and economic characteristics of the 28 countries that were validated for maternal and neonatal tetanus elimination during 2011 – 2020

**Country**	**2020 Total population (× 1000) **[11]	**2020 Surviving infant population (× 1000) **[11]	**2020 Pregnant women population (× 1000) **[11]	**Economic status **[15]
Burkina Faso	21,478	958	1,194	LIC
Cameroon	26,153	925	943	LMIC
Cambodia	16,609	363	368*	LMIC
Chad	16,802	805	925	LIC
China	1,400,050	14,650	ND	UMIC
Cote d'Ivoire	27,305	1,143	1,218	LMIC
Democratic Republic of Congo	112,578	4,503	4,503	LIC
Equatorial Guinea	1,146	28	40	UMIC
Ethiopia	101,767	3,424	3,421	LIC
Gabon	2,210	77	79	UMIC
Ghana	30,928	1,239	1,239	LMIC
Guinea Bissau	1,886	69	84	LIC
Haiti	11,743	264	328	LIC
India	1,356,000	27,338	30,062	LMIC
Indonesia	271,052	4,735	5,221	MIC/UMIC
Iraq	39,310*	1,079	ND	UMIC
Kenya	49,343	1,507	1,571	LMIC
Lao Peoples’ Democratic Republic	7,231	159	162*	LMIC
Liberia	4,461	191	205	LIC
Madagascar	27,301	963	1,228	LIC
Mauritania	4,173	155	185	LMIC
Niger	22,753	967	1,209	LIC
Philippines	108,877	2,123	2,123	LMIC
Senegal	16,705	578	603	LMIC
Sierra Leone	8,100	324	356	LIC
Tanzania	57,637	2,289	2,289	LIC/LMIC
Timor–Leste	1,299	41	41	LMIC
Uganda	41,583	2,016	2,079	LIC
Total	3,730,368	72,913	61,676	

*LIC* low-income country, *LMIC* lower middle-income country, *MIC* middle-income country, *UMIC* upper middle-income country, *ND* No data

### Maternal and neonatal tetanus elimination sustainability performance indicators

All 28 countries reported DTP3 coverage in 2020, and 7 (26%) attained ≥ 90% national coverage for DTP3. China had the highest coverage (99%) while Haiti reported the lowest (51%) DTP3 coverage. For the protection of women and infants against tetanus through immunization, ≥ 80% TTCV2 + coverage was achieved in 4 (16%) of 26 countries (no data for China and Kenya), while PAB coverage ≥ 80%, a more reliable estimate for tetanus protection than TTCV2 + , was attained in 23 (85%) of 27 countries (no data for China) (Table 2). There was a wide disparity between TTCV2 + and PAB coverage within countries (Table 3). In Tanzania, there was 51 percentage point difference in TTCV2 + coverage between the highest (73%) and lowest (22%) performing regions. Similarly, the PAB coverage in Cameroon had 41 percentage point difference between the highest (91%) and lowest (50%) performing regions (Table 3).

**Table 2:** Core and surrogate Maternal and Neonatal Tetanus Elimination (MNTE) sustainability indicators, platforms for introducing Tetanus Toxoid-Containing Vaccines (TTCV) booster doses, and Gavi eligibility status in the 28 countries that were validated for MNTE elimination during 2011–2020

Country	Year validated for MNTE		MNTE sustainability indicators						TTCV booster dose in national vaccination schedules			TTCV booster platforms			Gavi support [16]
		DTP3 [11]	TTCV2 + [11]	PAB [12]	ANC1 [13, 14]	ANC4 [13, 14]	HFD [13, 14]	SBA [13, 14]	1st booster [11]	2nd booster, [11]	3rd booster [11]	SVP exists [11]	MCV2 Intro [11]	HPV Intro [11]	
Burkina Faso	2012	91%	69%	95%	ND	ND	ND	ND	No	No	No	No	Yes	No	Yes
Cambodia	2015	92%	77%	95%	95%	79%	83%	89%	No	No	No	No	Yes	No	Yes
Cameroon	2012	69%	62%	83%	87%	65%	65%	69%	No	No	No	No	No	No	Yes
Chad	2019	52%	74%	78%	ND	ND	ND	ND	No	No	No	No	No	No	Yes
China	2012	99%	ND	ND	ND	ND	ND	ND	Yes	Yes	No	No	Yes	No	No
Cote d'Ivoire	2013	80%	75%	86%	ND	ND	ND	ND	No	No	No	Yes	No	Yes	Yes
DRC	2019	57%	96%	85%	ND	ND	ND	ND	No	No	No	No	No	No	Yes
E/Guinea	2015	53%	36%	60%	ND	ND	ND	ND	No	No	No	No	No	No	No
Ethiopia	2017	71%	90%	90%	74%	43%	48%	50%	No	No	No	Yes	Yes	Yes	Yes
Gabon	2013	63%	43%	83%	ND	ND	ND	ND	No	No	No	No	No	No	No
Ghana	2011	94%	62%	90%	98%	89%	79%	79%	No	No	No	No	Yes	No	Yes
G/Bissau	2012	74%	44%	83%	ND	ND	ND	ND	No	No	No	No	No	No	Yes
Haiti	2016	51%	44%	80%	91%	67%	39%	42%	Yes	No	No	No	Yes	No	Yes
India	2015	91%	78%	90%	79%	51%	79%	81%	Yes	Yes	No	No	Yes	No	Yes
Indonesia	2016	77%	54%	85%	98%	77%	74%	91%	Yes	Yes	Yes	Yes	Yes	Yes	No
Iraq	2013	74%	42%	73%	ND	ND	ND	ND	Yes	Yes	No	No	Yes	No	No
Kenya	2018	89%	ND	88%	ND	ND	ND	ND	No	No	No	No	Yes	Yes	Yes
Lao PDR	2013	79%	43%	93%	ND	ND	ND	ND	No	No	No	No	Yes	No	Yes
Liberia	2011	65%	20%	90%	98%	87%	80%	84%	No	No	No	No	Yes	Yes	Yes
Madagascar	2014	68%	52%	75%	ND	ND	ND	ND	No	No	No	No	No	No	Yes
Mauritania	2015	71%	31%	83%	85%	38%	70%	72%	No	No	No	No	No	No	Yes
Niger	2016	81%	71%	83%	ND	ND	ND	ND	No	No	No	No	Yes	No	Yes
Philippines	2015	71%	39%	91%	94%	87%	78%	84%	No	No	No	Yes	Yes	No	No
Senegal	2011	91%	68%	95%	98%	56%	80%	74%	No	No	No	Yes	Yes	Yes	Yes
Sierra Leone	2013	91%	95%	93%	98%	79%	83%	87%	No	No	No	No	Yes	No	Yes
Tanzania	2012	86%	100%	91%	98%	51%	63%	64%	No	No	No	Yes	Yes	Yes	Yes
Timor–Leste	2012	86%	34%	83%	84%	77%	49%	57%	Yes	Yes	No	Yes	Yes	No	Yes
Uganda	2011	89%	65%	83%	97%	60%	73%	74%	No	No	No	Yes	No	Yes	Yes

*E/Guinea* Equatorial Guinea, *G/Bissau* Guinea Bissau, *Lao PDR* Lao People’s Democratic Republic, *MNTE* maternal and neonatal tetanus elimination, *TTCV* tetanus toxoid containing-vaccines, *TTCV2* + at least two doses of tetanus toxoid containing-vaccines, *DTP3* third dose of diphtheria-tetanus-pertussis vaccine, *PAB* protection at birth against tetanus, *ANC1* one antenatal care visit, *ANC4* four antenatal care visits, *HFD* health facility delivery, *SBA* skilled birth attendance, *SVP* school vaccination program, *MCV2 intro* introduction of second dose of measles containing vaccines, *HPV intro* introduction of human papillomavirus vaccine, *Gavi* Gavi, the Vaccine Alliance *ND* No data

**Table 3:** Minimum and maximum coverage of maternal and neonatal tetanus elimination indicators by country second administrative level division (region / state / province) for: 1) at least two doses of tetanus toxoid-containing vaccines (TTCV2 +); 2) protection at birth against tetanus (PAB); 3) skilled birth attendance (SBA) and, 4) health facility delivery (HFD) in the 15 countries with available DHS / MICs data that were validated for maternal and neonatal tetanus elimination (MNTE) during 2011– 2020

Country	TTCV2 + [13, 14]		PAB [13, 14]		SBA [13, 14]		HFD [13, 14]	
	Min	Max	Min	Max	Min	Max	Min	Max
Cambodia	45%	80%	72%	98%	54%	98%	51%	97%
Cameroon	35%	75%	50%	91%	47%	99%	37%	99%
Ethiopia	ND	ND	ND	ND	26%	96%	23%	95%
Ghana	48%	68%	70%	82%	59%	92%	59%	92%
Haiti	58%	72%	69%	84%	28%	61%	26%	59%
India	57%	96%	64%	97%	61%	98%	51%	98%
Indonesia	33%	37%	56%	59%	86%	96%	60%	88%
Liberia	66%	91%	70%	92%	51%	97%	62%	96%
Mauritania	ND	ND	30%	65%	37%	99%	45%	98%
Philippines	45%	61%	66%	89%	34%	98%	28%	98%
Senegal	31%	59%	76%	91%	57%	92%	76%	95%
Sierra Leone	48%	95%	58%	97%	64%	98%	61%	97%
Tanzania	22%	73%	81%	97%	42%	96%	40%	94%
Timor-Leste	42%	80%	55%	89%	23%	85%	15%	83%
Uganda	46%	75%	71%	94%	58%	96%	56%	94%

*TTCV2* + at least two doses of tetanus toxoid-containing vaccines, *PAB* protection at birth against tetanus, *SBA* skilled birth attendance, *HFD* health facility delivery, *Min* minimum, *Max* maximum, *ND* no data

Of the 15 countries with available DHS/MICs data, ANC1 coverage was generally high with 13 (87%) of 15 countries attaining ≥ 80%; it ranged from 98% in Ghana, Indonesia, Liberia, Senegal, Sierra Leone, and Tanzania to 74% in Ethiopia. Only 3 (20%) of 15 countries attained ≥ 80% coverage for ANC4, which ranged between 89% in Ghana to 38% in Mauritania (Table 2). Among these 15 countries, 10 (67%) countries attained ≥ 70% SBA and HFD coverage. SBA coverage ranged from 91% in Indonesia to 42% in Haiti, and HFD coverage ranged from 83% in Cambodia and Sierra Leone to 39% in Haiti (Table 2). In ten (36%) of 28 countries, both PAB and SBA coverage figures were above the sustainability threshold (≥ 80% PAB and ≥ 70% SBA). In Ethiopia, Haiti, Tanzania, and Timor-Leste, even though SBA coverage was < 70%, PAB coverage was ≥ 80%. In Chad, Equatorial Guinea, Iraq, and Madagascar where the PAB and TTCV2 + were below the sustainability thresholds, lack of post-validation DHS or MICS did not allow us to understand if women and infants were protected through SBA or HFD (Table 2).

Disparities in sub-national level performance were also noted for all 15 countries (Table 3). In Ethiopia, for example, subnational coverage for SBA ranged from 26 to 96%, while HFD coverage ranged from 23 to 95%, with differences of 70 and 72 percentage points respectively.

### Protection against tetanus across the life-course

In terms of introduction of TTCV booster doses at the ages of 12–23 months, 4–7 years and 9–15 years, six (21%), five (21%), and one (4%) of the 28 countries reported having the first, second and third TTCV booster doses in their immunization schedule, respectively. Indonesia was the only country that provided all three booster doses. Of the eight countries that had a school vaccination programs (SVP), only two (Indonesia and Timor-Leste) reported providing a TTCV booster dose at school entry (Table 2). Comparing TTCV booster dose introduction to introduction of other vaccines provided beyond the first year of life, six of the 18 (33%) countries providing MCV2 also provided the first TTCV booster dose (both vaccines are given during the second year of life); and of the eight countries that provided HPV vaccine, only one (13%) reported providing the 3^rd^ booster dose of TTCV. India, Haiti, and Timor-Leste (LICs and LMICs) were the only Gavi supported countries to introduce TTCV booster doses, though we have no evidence that Gavi resources were used. Indonesia, China, and Iraq —all MICs or UMICs and non-Gavi eligible— have also introduced TTCV booster doses. Equatorial Guinea, an UMIC, had not yet introduced any TTCV booster dose (Tables 1 and 2).

### Barriers to health care services among women

In the DHS or MICS, socioeconomic and demographic barriers to accessing health care by women, which may also affect access to maternal and newborn care, included: 1) need for permission to seek healthcare, mostly from their husbands (range: 5% in Uganda to 35% in Cameroon and Timor-Leste, 2) getting money for treatment (range: 15% in Indonesia to 73% in Haiti), 3) long distance to reach health facilities (range: 11% in Indonesia to 50% in Ethiopia), and 4) not wanting to go alone (range: 13% in Senegal to 45% in Cambodia). Women who reported facing at least one obstacle in accessing healthcare ranged from 36% in Indonesia to 76% in Haiti (Table 4).

**Table 4:** Percentage of women age 15–49 who reported that they have serious problems in accessing health care for themselves when they are sick, by type of problem in the 14 countries* that were validated for maternal and neonatal tetanus elimination during 2011 –2020 and had a Demographic and Heath Survey (DHS) conducted post-MNTE validation

Country	Getting permission to go for treatment	Getting money for treatment	Health facility too distant	Not wanting to go alone	At least one problem accessing health care
Cambodia	21%	64%	35%	45%	75%
Cameroon	35%	67%	40%	28%	72%
Ethiopia	32%	55%	50%	42%	70%
Ghana	7%	48%	24%	14%	57%
Haiti	9%	73%	37%	20%	78%
India	ND	ND	ND	ND	ND
Indonesia	6%	15%	11%	26%	36%
Liberia	14%	36%	28%	19%	45%
Philippines	9%	45%	22%	21%	54%
Senegal	12%	43%	21%	13%	52%
Sierra Leone	24%	67%	44%	22%	72%
Tanzania	14%	50%	42%	30%	66%
Timor-Leste	35%	38%	46%	41%	60%
Uganda	5%	45%	37%	21%	59%

Data source: Demographic and Health Survey (DHS) https://dhsprogram.com/ [13] MNTE: maternal and neonatal tetanus elimination* The preliminary report of the 2019/2020 DHS for Mauritania (Mauritanie Enquête Démographique et de Santé (EDS) 2019–2020 [PR125] (dhsprogram.com), the only post-validation DHS does not include information on problems accessing health care

*ND* No data

### Performance of MNTE sustainability indicators in five countries that conducted MNTE post-validation assessments

During 2011‒2020, only three countries (Cameroon, Indonesia, and Timor-Leste) that have been validated for MNTE during that timeframe have conducted the WHO-recommended post-MNTE validation assessments. All countries attained core MNTE sustainability indicators (≥ 80% TTCV2 + and ≥ 70% SBA), except in Indonesia where the TTCV2 + coverage fell short of the threshold at 54% but PAB coverage was ≥ 80% (Table 2). The main recommendations from the assessments for all 3 countries included: 1) Strengthening NT surveillance, 2) improving health workers’ ability to correctly compute TTCV2 + and PAB coverage, 3) conducting periodic NT risk assessment for corrective actions, and 4) overall strengthening of maternal, newborn, child, and adolescent health. Cameroon was the only country that conducted the WHO recommended NT risk analysis and used the outcomes of the assessment for corrective action by targeting WRA in high-risk districts with two rounds of TTCV SIAs to maintain MNTE.

## Discussion

Most of the countries that were validated for the elimination of MNT during 2011‒2020 appear to be sustaining their elimination status. PAB coverage was ≥ 80% in almost all countries (except Chad, Equatorial Guinea, Iraq, and Madagascar). However, analysis at the subnational level reveals wide discrepancies in MNTE indicators at the second subnational level (region/provinces/states).

### TTCV infant series and childhood / adolescent booster doses

The majority of the 28 countries that have been validated for MNTE during 2011–2020, did not achieve ≥ 90% DTP3 coverage. The primary infant DTP series provides short-term protection and primes the immune system for the uptake of TTCV booster doses. A study on the barriers to childhood immunization in sub-Saharan Africa identified amongst others, parents’ or caretakers’ barriers that included lack of knowledge of immunization, distance to access point, financial deprivation, lack of family support, and distrust in vaccines and immunization programs. Health system barriers cited by healthcare providers included limited human resources and inadequate infrastructures to maintain the cold chain and inadequate supply of vaccines [17]. Scoping reviews of grey and peer reviewed literatures on reasons for non or under-vaccination of children identified issues related to the quality of immunization services offered, parental knowledge and attitudes, and household incomes [18–20]. To adequately sustain their MNTE status, countries need to address their specific health system barriers and implement existing strategies such the Reach Every District (RED) approach [21], periodic intensification of routine immunization (PIRI), [22] and country specific strategies to identify and vaccinate children focusing on “zero dose” children and communities [23].

Very few countries that have been validated for MNTE during the past 10 years have incorporated the recommended TTCV booster doses to sustain MNTE and promote protection against tetanus across the life course. Gavi’s and other immunization stakeholders’ support to MCV2 and HPV delivery has resulted in increased uptake of these vaccines in LICs and LMICs and is accelerating progress towards attaining the goals of the global initiatives associated with these vaccines. While tetanus does not have the outbreak potentials of the other vaccine-preventable diseases, maternal and neonatal tetanus affects the most marginalized and deprived segment of populations, and diphtheria outbreaks have been reported in several of those countries because of lack of booster doses (TTCV booster also include diphtheria boosters)[24]. In recognition of the inequitable access to health care that MNT represents and the need to deliver vaccines along a life-course, Gavi had planned to at least support the operational costs for the introduction of TTCV booster doses through its 2018 Vaccine Investment Strategy (VIS). However, the COVID19 pandemic has caused delays in this Gavi support [25]. Delivering the recommended three TTCV booster doses can be easily integrated with 1) MCV2 during second year of life, 2) school vaccination program at school entry and, 3) HPV vaccine during late childhood and adolescence. In addition, lifecourse vaccination and integration are key strategies in the Immunization Agenda 2030. With the high rates of school attendance in middle and upper-middle income countries and the increasing rates of school attendance (especially for primary school) in LICs and LMICs, for both boys and girls [26], significant efficiency gain, and shared benefits could accrue by sharing operational costs if schools with SVP and/or HPV vaccination programs were to offer Td booster doses to males and females during the same visits. Targeting boys with TTCV booster doses will allow for life-long protection against tetanus as well as diphtheria, while addressing the current gender imbalance between males and females in protection against tetanus [27, 28].

### Screening pregnant women at ANC visit to deliver appropriate doses of TTCV

ANC visits provide pregnant women opportunities to be vaccinated against tetanus and to receive health education on the importance of SBA and HFD as well as appropriate cord care practices. Coverage with at least one ANC visit was high in almost all 28 countries. However further improvement in ANC4 visit coverage and implementation of the WHO recommendation of at least eight ANC contacts during pregnancy will further enhance opportunities for vaccination and clean deliveries [29].

The wide disparity in coverage between TTCV2 + and PAB in assessing protection against tetanus in nearly all countries reflects the limited ability of health workers in using the assessment methods, especially in LICs and LMICs, where the calculation of PAB has been limited at the health facility level during ANC visits. The PAB assessment method takes into consideration, TTCV doses provided during infancy, booster doses, and doses administered during ANC visits, outreach and mobile sessions, and during SIAs [8]. In countries that have conducted Meningitis A (Men A) SIAs, the doses also count towards PAB, since tetanus toxoid is used as the carrier protein for the Men A polysaccharide, among other conjugate vaccines [1, 30, 31]. WHO estimates PAB coverage for all its member states annually, and in most cases these estimates are higher than the countries’ reported TTCV2 + coverage. Countries that have institutionalized the PAB assessment method have recorded improved coverage in their protection against tetanus by addressing the underestimation caused by TTCV2 + coverage method alone [32].

Training of health workers and availability of data collection tools (home-based records and health facility registers) could significantly improve the computation of tetanus protection through vaccination. Technical support to countries to use the PAB method could eventually help address the underestimation of true level of protection against tetanus among pregnant women.

### Access to skilled birth attendants and health facility delivery

Access to skilled birth attendants (doctors, nurses, and midwives) and health facilities during childbirth significantly improves the chances that deliveries will be clean and performed under hygienic conditions, and that appropriate hygienic umbilical cord care practices will take place once mothers are discharged from the health facilities. Despite the high percentage of countries with SBA and HF deliveries among the 15 countries with available DHS/MICs data, concerns exist about the 5 (33%) LICs and LMICs that had < 70% SBA or HFD, and the 13 countries with no DHS/MICs data post-MNTE validation to assess SBA and HFD coverage. The low SBA and HFD coverage in several countries could be attributed to the weak health systems, socioeconomic, infrastructural, and cultural factors that limit access by women to essential health care including, maternal and newborn and child health interventions. Traditional birth attendants (TBA) and deliveries assisted by female family members are still being used especially in remote rural areas. A study of the determinant of SBA delivery in Eastern African countries concluded that maternal age, women and husband education, wealth index, antenatal care visit, multiple gestations, parity, accessing health care, and residence were major determinants of skilled attendant delivery [33]. Strategies to overcome these barriers, most of which are outlined in several WHO guidelines, have been found to improve maternal and newborn outcomes. These include educating pregnant women, partners, and families on the importance of SBA and clean deliveries, and building sufficient and competent health workforces that are supported by adequate resources, safety culture, and safe working environments [34, 35].

### Post-validation assessments to monitor MNTE sustainability

Since the validation of MNTE, only five countries (three during 2011‒2020) have implemented the WHO recommended periodic NT risk analysis and post-validation assessments for corrective actions. This low uptake could be attributed to complacency, competing priorities, and deprioritizing MNTE once countries have been validated. Complacency leads to the risk of re-emergence of maternal and neonatal tetanus deaths. Hence, countries need to conduct periodic NT risk analysis and seize the opportunities to integrate post-MNTE validation assessments with reviews of the immunization and other health program. Tracking immunization program reviews planned by countries and advocating for the inclusion of MNTE post-validation assessments could help replace the time consuming and resource intensive standalone post-validation assessment, which are usually not attractive to stakeholders, and could increase compliance by countries.

### Limitations

The data for this study were mostly obtained from household surveys which were conducted during different years and covered varying periods in each country. Older surveys contain data that may no longer reflect the current situations in the countries. Only half of the countries had recent available DHS/MICS data to assess uptake of maternal and reproductive services. These severely limited the comparison of performance of those indicators across the countries. A better understanding of the performance of MNTE is based on district level analyses, and as the data for our study were mostly at the national level, it was difficult to understand the real country performance for MNTE. In addition, lack of adequate data on neonatal tetanus cases and deaths especially at the district level limited our ability to assess this indicator. Countries need to improve NT surveillance as it is one of the important indicators for MNTE validation and sustainability [4].

## Conclusions

Since neonatal tetanus elimination is defined as < 1 NT case per 1000 live births in each district per year, countries need to address those gaps in immunization coverage and ensure access to safe delivery at the lowest level to sustain MNTE. In LICs and MICs, scarcity of resources and fragile health systems continue to limit the uptake of DTP3 doses, life-course TTCV booster doses, and strengthening of maternal and newborn health care. The sub-optimal implementation of MNTE sustainability strategies could result in the reemergence of MNT as a public health concern in some of these countries. Joint planning, implementation, and monitoring of MNTE sustainability strategies by country immunization and maternal, newborn, child, and adolescent health programs will ensure that countries validated for MNTE maintain their status. New opportunities presented through various strategies, including the Immunization Agenda 2030 which includes MNTE as an endorsed vaccine preventable disease elimination target and promotes a lifecourse vaccination approach, could be leveraged by countries to sustain their MNTE status. These include school health programs, vaccine introductions in the second year of life, HPV introduction, zero dose agenda, integration of services, and life-course immunization delivery platforms. Once available, the planned Gavi Vaccine Investment Strategy (VIS) support for the introduction of TTCV booster doses will be a great opportunity to seize.

## Data Availability

The data supporting the results reported in this article can be found at the World Health Organization (WHO) Immunization Joint Reporting Forms (JRF). World Health Organization. 2021. https://immunizationdata.who.int/ and The DHS Program. Demographic and Health Surveys. https://www.dhsprogram.com/Countries/

## Abbreviations

ANC: Antenatal Care
ANC1: One Antenatal Care visit
ANC4: Four Antenatal Care Visits
C. tetanus: Clostridium tetani
DHS: Demographic and Health Survey
DTP: Diphtheria, Tetanus, Pertussis
DTP3: Third dose of Diphtheria, Tetanus, Pertussis
Gavi: Global Alliance for Vaccines and Immunization
HPV: Human Papillomavirus
HFD: Health Facility Delivery
JRF: Joint Reporting Form
LICs: Low-Income Countries
LMICs: Lower Middle-Income Countries
MCV2: Second dose of Measles-Containing Vaccine
Men A: Meningitis A
MICs: Middle-Income Countries
MICS: Multiple Indicator Cluster Survey
MNT: Maternal and Neonatal Tetanus
MNTE: Maternal and Neonatal Tetanus Elimination
NT: Neonatal Tetanus
PAB: Protection at Birth
PIRI: Periodic Intensification of Routine Immunization
RED: Reach Every District
SBA: Skilled Birth Attendance
SIAs: Supplementary Immunization Activities
SVP: School Vaccination Program
TBA: Traditional Birth Attendant
TTCV: Tetanus Toxoid Containing Vaccine
TTCV2 +: Two or more doses of Tetanus Toxoid-Containing Vaccine
UMICs: Upper Middle-Income Countries
UNICEF: United Nations Children Fund
VIS: Vaccine Investment Strategy
WHA: World Health Assembly
WHO: World Health Organization
WRA: Women of Reproductive Age

## References

[1] World Health Organization. Tetanus vaccines – February 2017: WHO position paper. Weekly Epidemiological Record 92 (6), 53 - 76. https://apps.who.int/iris/bitstream/handle/10665/254583/WER9206-53-76.pdf?sequence=1&isAllowed=y (Accessed 11 Oct 2021)

[2] World Health Organization. The perspective. Maternal and Neonatal Tetanus Elimination (MNTE). https://www.who.int/initiatives/maternal-and-neonatal-tetanus-elimination-(mnte) . (Accessed 11 Oct 2021)

[3] World Health Organization. Progress towards global MNT elimination. Maternal and Neonatal Tetanus Elimination (MNTE). World Health Organization 2020. https://www.who.int/initiatives/maternal-and-neonatal-tetanus-elimination-(mnte)/progress-towards-global-mnt-elimination. (Accessed 11 Oct 2021).

[4] World Health Organization. Neonatal Tetanus: Vaccine-preventable diseases surveillance standards: Updated September 5 2018. https://www.who.int/publications/m/item/vaccine-preventable-diseases-surveillance-standards-neonatal-tetanus (Accessed 11 Oct 2021)

[5] World Health Organization. The Strategies. Maternal and Neonatal Tetanus Elimination. https://www.who.int/initiatives/maternal-and-neonatal-tetanus-elimination-(mnte)/the-strategies. (Accessed 11 Oct 2021)

[6] Yusuf N, Raza AA, Blanc DC (2021). Progress and barriers towards maternal and neonatal tetanus elimination in the remaining 12 countries: a systematic review. Lancet Global Health.

[7] Njuguna HN, Yusuf N, Abid Raza A, Ahmed B, Tohme RA (2020). Progress Toward Maternal and Neonatal Tetanus Elimination — Worldwide, 2000–2018. MMWR Morb Mortal Wkly Rep.

[8] World Health Organization. Protecting all against tetanus: Guide to sustaining maternal and neonatal tetanus elimination (MNTE) and broadening tetanus protection for all. World Health Organization 2019. https://www.who.int/publications/i/item/protecting-all-against-tetanus (Accessed 11 October 2021)

[9] World Health Organization. Report of the SAGE Working Group on Maternal and Neonatal Tetanus Elimination and Broader Tetanus Prevention. https://www.who.int/immunization/sage/meetings/2016/october/1_Report_of_the_SAGE_Working_Group_on_Maternal_and_Neonatal_Tetanus_27Sep2016.pdf. (Accessed 11 Oct 2021)

[10] World Health Organization. The “high-risk” approach: the WHO-recommended strategy to accelerate elimination of neonatal tetanus. World Health Organization 1996. https://apps.who.int/iris/handle/10665/229665. (Accessed 11 Oct 2021)

[11] World Health Organization. Immunization Joint Reporting Forms (JRF). World Health Organization. 2021. https://immunizationdata.who.int/ (Accessed 11 Oct 2021.

[12] World Health Organization. The Global Health Observatory. Neonates protected at birth against neonatal tetanus (PAB) (%). World Health Organization.2020 https://www.who.int/data/gho/data/indicators/indicator-details/GHO/neonates-protected-at-birth-against-neonatal-tetanus-(pab)-(-). (Accessed 11 Oct 2021)

[13] The DHS Program. Demographic and Health Surveys. https://www.dhsprogram.com/Countries/ (Accessed 11 Oct 2021)

[14] UNICEF. Multiple Indicator Cluster Surveys (MICS). https://mics.unicef.org/surveys (Accessed 11 Oct 2021)

[15] The World Bank. Data. World Bank Country and Lending Group. The World Bank. 2020. https://datahelpdesk.worldbank.org/knowledgebase/articles/906519-world-bank-country-and-lending-groups . (Accessed 11 Oct 2021)

[16] Gavi. Accelerate the uptake and use of underused and new vaccines. The vaccine goal. Gavi, the Vaccine Alliance. https://www.gavi.org/our-alliance/strategy/phase-3-2011-2015/vaccine-goal (Accessed 11 Oct 2021)

[17] Bangura JB, Xiao S, Qiu D, Ouyang F, Chen L (2020). Barriers to childhood immunization in sub-Saharan Africa: A systematic review. BMC Public Health..

[18] Rainey JJ, Watkins M, Ryman TK, Sandhu P, Bo A, Banerjee K (2011). Reasons related to non-vaccination and under-vaccination of children in low- and middle-income countries: findings from a systematic review of the published literature, 1999–2009. Vaccine.

[19] Favin M, Steinglass R, Fields R, Banerjee K, Sawhney M (2012). Why children are not vaccinated: a review of the grey literature. Int Health.

[20] Ntenda PAM (2019). Factors associated with non-and-under-vaccination among children aged 12–23 months in Malawi. A multinomial analysis of the population-based sample. Paediatr Neonatology..

[21] WHO Regional Office for Africa. 2017: Reaching Every District (RED)2017 Edition. A guide to increasing coverage and equity in all communities in the African Region. https://apps.who.int/iris/bitstream/handle/10665/260112/9789290233954-eng.pdf?sequence=1&isAllowed=y. (Accessed 11 Oct 2021)

[22] World Health Organization. Periodic Intensification of Routine Immunization. Lessons Learned and Implications for Action. Using PIRI as an opportunity to strengthen routine immunization. Page 32. World Health Organization. 2009 https://www.who.int/immunization/programmes_systems/policies_strategies/piri_020909.pdf. (Accessed 11 Oct 2021)

[23] Gavi. The Zero-Dose Child: Explained. Gavi, the Vaccine Alliance. 2021. The Zero-Dose Child: Explained | Gavi, the Vaccine Alliance. (Accessed 11 Oct 2021)

[24] Clarke KEN, MacNeil A, Hadler S, Scott C, Tiwari TSP, Cherian T (2019). Global epidemiology of diphtheria, 2000–2017. Emerg Infect Dis.

[25] Gavi. Vaccine Investment Strategies. Report to the Board November 2018. Board Document Template (gavi.org). (Accessed 11 Oct 2021)

[26] The World Bank. Institute for Statistics. School enrolment, primary (% gross) Lower middle income. The World Bank. 2020. https://data.worldbank.org/indicator/SE.PRM.ENRR?locations=XN-XM (Accessed 11 Oct 2021).

[27] Dalal S, Samuelson J, Reed J, Yakubu A, Ncube B, Baggaley R (2016). Tetanus disease and deaths in men reveal need for vaccination. Bull World Health Organ.

[28] Scobie HM, Patel M, Martin D (2017). Tetanus Immunity Gaps in Children 5–14 Years and Men >/= 15 Years of Age Revealed by Integrated Disease Serosurveillance in Kenya, Tanzania, and Mozambique. Am J Trop Med Hyg.

[29] World Health Organization. WHO recommendations on antenatal care for a positive pregnancy experience. World Health Organization. 2017. https://www.who.int/publications/i/item/9789241549912 . (Accessed 11 Oct 2021).

[30] Basta NE, Borrow R, Berthe A, Onwuchekwa U, Dembele ATE, Almond R (2015). Higher tetanus toxoid immunity 2 years after PsA-TT introduction in Mali. Clin Infect Dis.

[31] Borrow R, Tang Y, Yakubu A, Kulkarni PS, Laforce FM (2015). MenAfriVac as an antitetanus vaccine. Clin Infect Dis.

[32] World Health Organization. Protection-at-birth (PAB) method, Tunisia: Monitoring tetanus toxoid coverage and avoiding missed opportunities for tetanus toxoid vaccination. World Health Organization. 2000. Weekly Epidemiological Record (WER). 75 (25), 203 – 206. https://apps.who.int/iris/handle/10665/231194. (Accessed 11 Oct 2021)

[33] Tessema ZT, Tesema GA (2020). Pooled prevalence and determinants of skilled birth attendant delivery in East Africa countries: a multilevel analysis of Demographic and Health Surveys. Ital J Pediatr.

[34] World Health Organization. (2020). Human resource strategies to improve newborn care inhealth facilities in low- and middle-income countries. World Health Organization. https://apps.who.int/iris/handle/10665/336677. (Accessed 11 Oct 2021).

[35] World Health Organization. Making Pregnancy Safer. Global Action for Skilled Attendants for Pregnancy. 2002. https://www.unscn.org/layout/modules/resources/files/Making_pregnancy_safer_the_critical_role.pdf. (Accessed 03 Nov 2021)

